# Cross‐sectional study of psychosocial well‐being among lesbian, gay, bisexual, and heterosexual gynecologic cancer survivors

**DOI:** 10.1002/cnr2.1461

**Published:** 2021-05-31

**Authors:** Alexandra Schefter, Lauren Thomaier, Patricia Jewett, Katherine Brown, Ashley E. Stenzel, Anne Blaes, Deanna Teoh, Rachel I. Vogel

**Affiliations:** ^1^ Department of Obstetrics, Division of Gynecologic Oncology Gynecology and Women's Health, University of Minnesota Minneapolis Minnesota USA; ^2^ Department of Medicine, Division of Hematology and Oncology University of Minnesota Minneapolis Minnesota USA; ^3^ Program in Health Disparities Research, Department of Family Medicine & Community Health University of Minnesota Medical School Minneapolis Minnesota USA

**Keywords:** disparities, emotional health, gynecologic cancers, quality of life, sexual minority, sexual orientation

## Abstract

**Background:**

Delays in care and increased risk for mental health diagnoses put individuals identifying as a sexual minority with cancer at risk for decreased quality of life.

**Aim:**

To assess psychosocial health among sexual minority gynecologic cancer survivors, we compared self‐reported quality of life and psychosocial measures between individuals diagnosed with gynecologic cancers identifying as lesbian/gay/bisexual (LGB) and heterosexual.

**Methods and Results:**

English‐speaking adults with gynecologic cancers were invited to participate in an ongoing cohort survey study. Quality of life and psychosocial measures included the Functional Assessment of Cancer Therapy‐General, Distress Thermometer (distress), Patient Health Questionnaire‐8 (depression), General Anxiety Disorder‐7 (anxiety), and Post‐traumatic Stress Disorder Checklist for DSM‐5 (post‐traumatic stress disorder; PTSD). Measures were compared by self‐reported sexual orientation (heterosexual vs. LGB) using descriptive statistics (frequencies and means) and linear and logistic regression models, adjusting for college education.

Of 814 patients invited, 457 enrolled (56.1%) and 401 (92.6%) completed the survey and provided information on their sexuality. All but one self‐identified as cisgender women and 22 (5.5%) as LGB. LGB participants were more likely to have completed college (68.2% vs. 40.1%, *p* = .009) but were otherwise similar across demographic and clinical characteristics. Quality of life and distress scores were similar between groups. LGB participants, compared to heterosexual, reported higher rates of depression (31.8% vs. 10.6%, adjusted odds ratio [OR] = 4.1 [95% confidence interval [CI]: 1.6–11.0], *p* = .004), anxiety (25.0% vs. 7.1%, adjusted OR = 5.4 [95% CI: 1.7–16.7], *p*= .004), and PTSD (13.6% vs. 3.5%, adjusted OR = 4.2 [95% CI: 1.1–16.3], *p* = .04).

**Conclusion:**

LGB participants reported poorer emotional health following a gynecologic cancer diagnosis than heterosexual participants. Our data suggest this population may need additional resources and support during and after their cancer diagnosis. Future work is needed to identify additional risk factors and the underlying sources of these disparities in order to improve patient care and wellness in this population.

## 1 INTRODUCTION

Treatment innovations for cancers have resulted in improved clinical outcomes and a growing population of cancer survivors in the United States. As a result, the long‐term negative effects associated with treatment and disease have become an increasing public health concern. While there is a growing body of literature focused on cancer survivorship, few prospective studies exist focused on quality of life (QOL) for gynecologic cancer survivors, and there is a paucity of existing research surrounding specifically sexual minority populations of gynecologic cancer survivors.^1^

Individuals who belong to societally marginalized populations are at increased risk for adverse health outcomes, as described in The Health Equity Promotion Model and Minority Stress Theory.^2^, ^3^ Discriminatory or stigmatized experiences within one's family and community, re‐experienced in clinical settings may impact one's mental and physical health. Multiple studies suggest that individuals belonging to sexual minority populations have a higher prevalence of gynecologic cancers due to increased prevalence of risk factors such as smoking and obesity, as well as decreased likelihood of cancer screening.^4^, ^5^, ^6^, ^7^, ^8^, ^9^, ^10^, ^11^, ^12^, ^13^, ^14^ Furthermore, disparities in the rates of depression, anxiety, and substance abuse have previously been shown between sexual minority individuals and their heterosexual counterparts. Rates of mental health disorders are estimated to be approximately 2–3 times higher in the sexual minority population compared to that of the general population.^15^, ^16^ Both the increased rate of substance abuse and increased rates of mental health disorders in this population are likely influenced by prejudice and discrimination.^17^

Despite a higher prevalence of cancer‐related risk factors, individuals belonging to a sexual minority group have greater delays to diagnosis and treatment. Decreased rates of health insurance, poorer access to healthcare and financial barriers leading to unmet medical care have been suggested as inciting factors.^6^, ^17^, ^18^, ^19^ Furthermore, there continues to be a need for increased medical education among practitioners and nursing staff on the topic of sexual minority health, potentially contributing to disparities in quality of care received by members of this community.^20^, ^21^, ^22^, ^23^, ^24^, ^25^, ^26^, ^27^, ^28^ Lack of access to adequate healthcare and insurance, stigmatization and increased prevalence for mental health diagnoses prior to cancer diagnosis put this population of cancer survivors at particular risk for poorer outcomes and QOL following a cancer diagnosis. However, evidence on psychosocial outcomes among this population of gynecologic cancer survivors is limited with inconsistent findings.^29^, ^30^, ^31^ Given the potential impact of gynecologic cancer on psychosocial health outcomes in general, and the greater prevalence of clinical risk factors among sexual minority populations specifically, further identifying disparities among subgroups of survivors is necessary to identify highest risk individuals for worse outcomes.^32^, ^33^ We sought to compare self‐reported QOL and psychosocial measures between heterosexual and sexual minority gynecologic cancer survivors.

## 2 METHODS

The Gynecology Oncology‐Life after Diagnosis (GOLD) cohort study prospectively assesses QOL and long‐term social, emotional, and physical sequelae of cancer in gynecologic cancer survivors. Study methods have been published elsewhere.^34^ Briefly, English‐speaking individuals, 18 years or older, diagnosed with gynecologic cancer (ovarian, cervical, endometrial, vaginal, or vulvar) at the University of Minnesota were identified using electronic medical records. Individuals were recruited in clinic (March 2017–March 2020) and via mail (June–October 2018) to participate in the cohort. Time since diagnosis was not an eligibility criterion; however, most were invited after completion of initial treatment. Participants completed online or paper surveys. The GOLD study was approved by the Institutional Review Board at the University of Minnesota. Participants provided signed informed consent and Health Insurance Portability and Accountability Act forms for study participation and abstraction of clinical data from the electronic health record.

The primary outcomes for this exploratory cross‐sectional analysis were self‐report measures of QOL and emotional health at the time of study entry. Cancer‐related QOL was measured using the Functional Assessment of Cancer Therapy‐General (FACT‐G); higher scores indicate greater QOL.^35^, ^36^ Cancer‐related distress was measured using the National Comprehensive Cancer Network Distress Thermometer, version 2.2016, with higher scores indicating greater distress; a score of 4 or greater was considered clinically relevant distress.^37^ Depression was measured using the Patient Health Questionnaire‐8 (PHQ‐8) score; higher scores indicate greater depression symptoms; a score of 10 or greater was considered potentially clinically relevant depression symptoms.^38^ Generalized anxiety was measured using the General Anxiety Disorder‐7 (GAD‐7), with higher scores indicating greater anxiety severity and a score of 10 or greater was considered potentially clinically relevant anxiety symptoms.^39^ Symptoms of post‐traumatic stress disorder (PTSD) were measured using the Post‐traumatic Stress Disorder Checklist for DSM‐5, with higher scores indicating greater PTSD symptoms and a score of 33 or greater considered potentially clinically relevant.^40^

Sexual orientation was self‐reported by participants (heterosexual, lesbian, gay, bisexual, or other). Due to the small sample size, participants who identified as lesbian, gay, bisexual were categorized as LGB for analysis purposes. No participants reported “other.”

Additional self‐reported data collected via the survey included demographic variables, which were categorized for analyses as follows: age (continuous, years), gender (male, female, transgender, other), race/ethnicity (non‐Hispanic white, other), education (no college degree, at least a college degree), income (annual household income <$50 000, $50 000–$99 999, ≥$100 000, prefer not to say), marital status (partnered/married, widowed/divorced/never married), employment status (part/full time, retired, unemployed), urbanicity (rural, urban‐based on Zoning Improvement Plan (ZIP) codes/Rural‐Urban Commuting Area (RUCA) codes^41^), and treatment status at time of study entry (receiving treatment for initial diagnosis, for progression/recurrence, not receiving treatment). Clinical data abstracted from the electronic medical record included primary cancer site, International Federation of Gynecology and Obstetrics (FIGO) stage at diagnosis, date of diagnosis, and treatments received for their cancer (chemotherapy, surgery, and/or radiation).

Patient‐reported QOL and psychosocial measures were compared by sexual orientation using descriptive statistics, chi‐squared tests, Fisher's exact tests, and *t*‐tests as appropriate. Due to the limited sample size, linear (continuous measures) and logistic (clinically relevant cutoffs) regression models were performed, adjusting for college education (yes/no) only. We ran two additional sensitivity analyses, adjusting for (1) time since diagnosis and (2) current treatment status. In a supplemental analysis, we reviewed the medical records of all LGB participants and of heterosexual participants with elevated anxiety or depression scores for mental health disorders diagnosed prior to their cancer. Individuals with missing outcome data were excluded from analyses related to just that outcome measure. Data were analyzed using SAS version 9.4 software (SAS Institute Inc., Cary, North Carolina), and *p*‐values less than .05 were considered statistically significant.

## 3 RESULTS

A total of 814 individuals were invited to participate in this study; 457 participants enrolled (56.1% total response rate; 252 of 590 (42.6%) via mail and 205 of 224 (91.5%) in clinic). Of these, 421 completed the survey and 401 (92.6%) provided information on their sexual orientation and were included in this analysis.

All participants self‐identified as cisgender women except one participant who identified as transgender and 22 participants (5.5%) self‐reported being LGB (14 (3.5%) gay/lesbian and 8 (2.0%) bisexual). Participants were on average 60.0 ± 11.0 years old and 2.1 ± 2.0 years from diagnosis. Most were non‐Hispanic white (97.4%) and had not completed college education (60.0%). The majority were diagnosed with ovarian (42.7%) or endometrial (39.6%) cancer, with the remaining 11.9% and 5.8% having cervical or vaginal/vulvar cancers, respectively. Demographic and clinical variables were generally similar between groups; however, LGB participants were more likely to have completed a college education (68.2% vs. 40.1%, *p* = .009; Table 1).

**TABLE 1:** Demographic and clinical characteristics by sexual orientation, *N* = 401

Variable	Heterosexual		Lesbian/Gay/Bisexual		*p*‐value
	(*N* = 379)		(*N* = 22)		
	*N*	Mean ± SD	*N*	Mean ± SD	
**Age at survey, years**	379	60.2 ± 10.8	22	57.8 ± 9.5	.32
**Charlson Comorbidity Index**	370	2.3 ± 1.8	22	1.7 ± 1.0	.12
	*N*	%	*N*	%	*p*‐value
**Race/Ethnicity**					.14
Other	10	2.7	2	9.1	
Non‐Hispanic white	367	97.4	20	90.9	
**Education**					.009
Less than college	226	60.0	7	31.8	
At least college	151	40.1	15	68.2	
**Marital status**					.19
Widowed/divorced/never married	150	40.3	12	54.6	
Married/partnered	222	59.7	10	45.5	
**Income**					.73
<$50 000	130	34.7	7	31.8	
$50 000–$99 999	128	34.1	8	36.4	
≥$100 000	75	20.0	6	27.3	
Prefer not to say	42	11.2	1	4.6	
**Employment**					.64
Yes—full or part time	190	50.4	13	59.1	
No	47	12.5	1	4.6	
Retired	140	37.1	8	36.4	
**Urban/rural residence**					.06
Rural	58	15.3	0	0.0	
Urban	321	84.7	22	100.0	
**Time since first diagnosis**					.89
<1 year	116	30.8	7	31.8	
1‐<2 years	107	28.4	5	22.7	
2‐<5 years	140	37.1	9	40.9	
≥5 years	14	3.7	1	4.6	
**Primary cancer type**					.96
Ovarian	162	42.7	10	45.5	
Cervical	45	11.9	3	13.6	
Endometrial	150	39.6	8	36.4	
Vaginal/Vulvar	22	5.8	1	4.6	
**Cancer stage**					.78
I or II	215	57.6	12	54.6	
III or IV	158	42.4	10	45.5	
**Surgery**					1.00
No	33	8.7	2	9.1	
Yes	346	91.3	20	90.9	
**Chemotherapy**					.72
No	135	35.6	7	31.8	
Yes	244	64.4	15	68.2	
**Radiation**					.58
No	279	73.6	15	68.2	
Yes	100	26.4	7	31.8	
**Treatment status at time of survey**					.14
Receiving initial treatment	28	7.6	4	18.2	
Receiving treatment for disease progression/recurrence	58	15.8	4	18.2	
Not currently receiving treatment	282	76.6	14	63.6	

Rates of potentially clinically relevant depression (31.8% vs. 10.6%, *p* = .003), anxiety (25.0% vs. 7.1%, *p* = .02), and PTSD (13.6% vs. 3.5%, *p* = .05) were significantly more common in LGB participants compared to heterosexual participants (Table 2). After adjusting for education, these differences remained: we observed higher rates of clinically relevant depression (adjusted OR = 4.1 [95% CI: 1.6‐11.0], *p* = .004), anxiety (adjusted OR = 5.4 [95% CI: 1.7‐16.7], *p* = .004), and PTSD (adjusted OR = 4.2 [95% CI: 1.1‐16.3], *p* = .04) among LGB participants. No significant differences in overall QOL and distress were observed. Results were similar after additionally adjusting for time since diagnosis and current treatment status.

**TABLE 2:** Psychosocial measures and outcomes by sexual orientation

	Heterosexual (*N* = 379)		Lesbian/Gay/Bisexual (*N* = 22)		*p*‐value
	*N*	Mean ± SD	*N*	Mean ± SD	
**Distress thermometer score**	357	2.8 ± 2.7	21	3.4 ± 3.1	.30
**FACT Quality of Life (QOL) total score** ^a^	375	83.3 ± 16.3	22	80.0 ± 22.7	.37
QOL—physical subscale score	377	22.8 ± 5.2	22	22.7 ± 5.5	.99
QOL—social subscale score	376	21.6 ± 5.8	22	20.4 ± 7.3	.35
QOL—emotional subscale score	378	18.6 ± 4.1	22	17.4 ± 5.4	.18
QOL—functional subscale score	378	20.3 ± 5.6	22	19.5 ± 7.5	.50
**PHQ‐8 Depression score** ^b^	377	4.2 ± 4.1	22	6.6 ± 6.5	.01
**PCL‐5 PTSD score** ^c^	377	8.5 ± 9.5	22	15.6 ± 17.0	.001
**GAD‐7 Anxiety score** ^d^	368	3.4 ± 3.7	20	5.7 ± 6.2	.01
	*N*	%	*N*	%	
**Distress symptoms** ^e^	115	32.2	9	42.9	.31
**Depression symptoms** ^e^	40	10.6	7	31.8	.003
**PTSD symptoms** ^e^	13	3.5	3	13.6	.05
**Anxiety symptoms** ^e^	26	7.1	5	25.0	.02

^a^ Functional Assessment of Cancer Therapy‐General (FACT‐G).

^b^ Patient Health Questionnaire‐8 (PHQ‐8).

^c^ Posttraumatic Stress Disorder Checklist for DSM‐5 (PCL‐5).

^d^ General Anxiety Disorder‐7 (GAD‐7).

^e^ Meeting symptoms threshold (distress thermometer 4+, PHQ‐8 10+, PCL‐5 33+, GAD‐7 10+).

Based on medical record review, 57.5% of heterosexual individuals who acknowledged depression symptoms at the time of study participation (survey) had previously been diagnosed with depression before their cancer, and 42.5% had depression symptoms at the time of their cancer diagnosis; these proportions were similar among LGB participants (57.1% and 57.1%, respectively), though numbers are small. Furthermore, 52.0% of heterosexual individuals who reported anxiety symptoms on the survey had an anxiety diagnosis before their cancer, and 32.0% had documented anxiety symptoms at the time of their cancer diagnosis; rates of anxiety prior to or at the time of diagnosis were lower among LGB individuals (20.0% and 20.0%, respectively). These data suggest that many, but not all, LGB participants had preexisting mental health disorders prior to their cancer, with a substantial number of participants developing such symptoms only after their cancer. Further, if we excluded those we identified as having depression and anxiety at the time of diagnosis, the conclusions were similar (LGB vs. heterosexual, depression adjusted OR = 5.2 [95% CI: 1.3–20.8], *p* = .02; and anxiety adjusted OR = 8.2 [95% CI: 2.3‐29.7], *p* = .001).

## 4 DISCUSSION

As the number of gynecologic cancer survivors increases, identification of risk factors associated with poorer outcomes and QOL is crucial for providing comprehensive care. Our findings suggest that individuals who identify as LGB are at risk for poorer emotional health following a gynecologic cancer diagnosis, specifically with regard to anxiety, depression, and PTSD. We did not observe significant differences in QOL by sexual identity group. While the FACT‐G captures overall emotional QOL and has demonstrated reliability for examining general QOL, the PHQ‐8, and GAD‐7 questionnaires assess specific mental health concerns and are accepted clinical tools with known cutoffs relevant for identifying depression and anxiety.^42^, ^43^, ^44^, ^45^

The American Cancer Society estimated that 107 290 individuals would be diagnosed with gynecologic cancers in 2020.^46^, ^47^ Conservatively, 1.2%–12% of the US population is estimated to identify as belonging to a sexual and/or gender minority group.^48^ Although epidemiologic data on the number of individuals with gynecologic cancers who identify as a sexual minority are lacking, extrapolating from the reports above, we can conservatively estimate that at least 1300 sexual and/or gender minority individuals will be diagnosed with a gynecologic cancer each year. Evidence suggests lesbian women are at increased risk for breast cancer,^9^ and bisexual individuals may be at increased risk for cervical cancer^4^; this is likely due at least in part to lower rates of cervical and breast cancer screening among the sexual minority population.^23^, ^48^, ^49^

Due to this population's potentially increased risk for cancer, it is imperative that studies inquire about, and report on, sexual orientation to better understand cancer survivorship issues. Furthermore, many studies represent limited cancer diagnoses, with the majority of the literature focused on survivorship issues related to breast, colorectal, and prostate cancer.^50^ Gynecologic cancers made up 5.6% of new cancer diagnoses and 5.1% of cancer deaths in women from 2013 to 2017.^47^ It is important to identify associated cancer survivorship issues, especially as these diagnoses may be intimately related to gender identity and sexual orientation.

There are many studies that suggest that sexual minority persons are at greater risk for poor mental health, with increased rates of depression, anxiety disorders, suicide attempts, and comorbid mental health conditions in general.^51^ These patients often describe interpersonal and structural barriers, lack of provider knowledge regarding their sexual orientation, and concerns about disclosure of sexual orientation.^52^ As previously mentioned, these stressors, discriminatory experiences, and microaggressions experienced with a societally marginalized identity may impact health.^2^ Even if the emotional health of individuals identifying as a sexual minority was poor before their cancer diagnosis, the frequent clinical contact that comes with a cancer diagnosis presents a window of opportunity to identify and address emotional health, which may also impact the individual's ability to cope with the diagnosis. The interplay between preexisting and cancer‐related factors should be the focus of future studies.

It has also been shown that patients with poor access to care describe worse emotional health.^31^ In a recent study, Boehmer et al, showed that sexual minority female cancer survivors had significantly lower access to care compared to their heterosexual counterparts, and this was associated with poor physical and mental QOL.^31^ The underlying risk of poor mental health, combined with lower access to care, and unease regarding provider knowledge and trust makes the sexual minority cancer survivor population at specific risk for poorer mental QOL. This is exacerbated by poor ascertainment of gender identity and sexual orientation as part of oncology care.^53^ Matthews, et al, recently conducted a survey study of sexual and gender minority cancer survivors.^54^ They found that only 21% of patients knew of sexual minority specific support groups, but interestingly, 81% of patients utilized these services when available. Increasing awareness of such resources among providers and nurse educators may in turn increase the accessibility to patients.

A recent survey of oncologists by Schabath, et al, found that while 65.8% of oncologists believed knowing gender identity was important, only 39.6% believed knowing sexual orientation was important.^23^ Another recent study identified that greater disparities may exist among rural versus urban women identifying as lesbian sexual orientation, with those in a rural setting reporting fewer recommendations for Pap/Human Papillomavirus (HPV) screening from their women's health provider, and both reporting less than 50% of providers inquiring about sexual orientation.^55^ Our study, along with the previously noted studies, highlights a large area for improvement for provider training and education in order to offer comprehensive care for cancer survivors. Furthermore, identifying healthcare disparities for the sexual minority population will help to develop tailored preventive treatment and survivorship services. While routinely collecting information on sexual orientation and gender identity in clinical care may in itself to some degree increase awareness of unique populations and their needs, future studies should be performed with the goal of identifying solutions to these disparities. There is an immediate need for research addressing sources of disparities as outlined by the Minority Stress Theory^3^ and Health Equity Promotion Model,^2^ such as multilevel interventions targeting policy‐, community‐, clinic‐, and provider‐level interventions to educate providers on the unique needs of sexual minorities, to reduce stigmatization and discrimination, and to increase the quality of healthcare delivery for sexual minority patients based on structural‐ and individual‐level strategies.

Limitations of this exploratory analysis include a small cohort size (including only one transgender participant), though the proportion of LGB participants is reflective of the population. We did not perform a power analysis prior to proceeding with the study. The sample size did not allow for adjustment for other factors, or the ability to examine different sexual minorities separately, or intersectionality of multiple social constructs that likely create unique and group‐specific experiences of discrimination and impact clinical care, such as race, ethnicity, socioeconomic class, or among specific sexual and gender minority subgroups (ie, lesbian, bisexual, gay, queer, transgender). The tools used to examine anxiety, depression, and distress should be further examined among sexual and gender minority individuals as the validity and reliability of these tools were likely established among largely cisgender, heterosexual populations. We also did not measure insurance status, which also may have been related to the outcomes. While we recognize the importance of addressing discrimination and stigmatization to reduce disparities, we did not assess these factors and were unable to address these factors in this study. Future studies should examine these factors that may lead to differences in psychosocial outcomes with intervention studies to guide reduction of disparities as outlined by the minority stress and health equity promotion models.^2^, ^3^ Participants in this study were diagnosed and treated for gynecologic cancers at a single academic institution in Minnesota and were primarily non‐Hispanic white, thus our findings may not be generalizable. Finally, given the nature of the study and nonresponse rate, there may be response bias.

## 5 CONCLUSION

Our data highlight that LGB gynecologic cancer survivors may need additional, specific, and alternative resources and support following a cancer diagnosis. This is exacerbated by the lack of policies for patient‐centered care of sexual minority populations in cancer centers.^23^, ^49^ Multilevel and multidisciplinary training on the specific needs of sexual minority patients should be mandatory for care providers. Previous studies suggest the need for education for primary care providers in order to ensure sexual minority patients are receiving comprehensive cancer prevention care including cervical cytology and HPV testing, and appropriate breast cancer screening. In addition, our study suggests the need for specialized oncologic training on nuanced mental health support for sexual minorities. Future research should focus on developing interventions based on best practices for optimizing care for the unique needs of sexual minority gynecologic cancer survivors.

## CONFLICT OF INTEREST

Teoh reports grants from KCI/Acelity and Tesaro/GSK, outside the submitted work; the other authors have no other conflicts to report.

## AUTHOR CONTRIBUTIONS

**Alexandra Schefter:** Data curation; writing‐original draft. **Lauren Thomaier:** Data curation; writing‐original draft; writing‐review & editing. **Patricia Jewett:** Formal analysis; methodology; writing‐review & editing. **Katherine Brown:** Data curation; project administration; writing‐review & editing. **Ashley Stenzel:** Formal analysis; writing‐original draft; writing‐review & editing. **Anne Blaes:** Conceptualization; funding acquisition; methodology; writing‐review & editing. **Deanna Teoh:** Conceptualization; data curation; methodology; supervision; writing‐review & editing. **Rachel Vogel:** Conceptualization; formal analysis; funding acquisition; methodology; project administration; supervision; writing‐review & editing.

## ETHICS STATEMENT

This study was approved by the Institutional Review Board at the University of Minnesota. Participants provided signed informed consent and Health Insurance Portability and Accountability Act (HIPAA) forms for study participation and abstraction of clinical data from the electronic health record.

## Data Availability

The data that support the findings of this study are available on request from the corresponding author. The data are not publicly available due to privacy or ethical restrictions.
